# PCPPI: a comprehensive database for the prediction of *Penicillium*–crop protein–protein interactions

**DOI:** 10.1093/database/baw170

**Published:** 2017-02-26

**Authors:** Junyang Yue, Danfeng Zhang, Rongjun Ban, Xiaojing Ma, Danyang Chen, Guangwei Li, Jia Liu, Michael Wisniewski, Samir Droby, Yongsheng Liu

**Affiliations:** 1 College of Food Science and Engineering, Hefei University of Technology, Hefei 230009, China; 2 Ministry of Education Key Laboratory for Bio-resource and Eco-environment, College of Life Science and State Key Laboratory of Hydraulics and Mountain River Engineering, Sichuan University, Chengdu 610064, China; 3 School of Information Science and Technology, University of Science and Technology of China, Hefei 230026, China; 4 School of Medical Engineering, Hefei University of Technology, Hefei 230009, China; 5 United States Department of Agriculture-Agricultural Research Service (USDA-ARS), Kearneysville, WV 25430, USA; 6 Agricultural Research Organization (ARO), The Volcani Center, 50250 Bet Dagan, Israel

## Abstract

*Penicillium expansum*, the causal agent of blue mold, is one of the most prevalent post-harvest pathogens, infecting a wide range of crops after harvest. In response, crops have evolved various defense systems to protect themselves against this and other pathogens. *Penicillium*–crop interaction is a multifaceted process and mediated by pathogen- and host-derived proteins. Identification and characterization of the inter-species protein–protein interactions (PPIs) are fundamental to elucidating the molecular mechanisms underlying infection processes between *P. expansum* and plant crops. Here, we have developed PCPPI, the *Penicillium*-Crop Protein–Protein Interactions database, which is constructed based on the experimentally determined orthologous interactions in pathogen–plant systems and available domain–domain interactions (DDIs) in each PPI. Thus far, it stores information on 9911 proteins, 439 904 interactions and seven host species, including apple, kiwifruit, maize, pear, rice, strawberry and tomato. Further analysis through the gene ontology (GO) annotation indicated that proteins with more interacting partners tend to execute the essential function. Significantly, semantic statistics of the GO terms also provided strong support for the accuracy of our predicted interactions in PCPPI. We believe that all the PCPPI datasets are helpful to facilitate the study of pathogen-crop interactions and freely available to the research community.

**Database URL**: http://bdg.hfut.edu.cn/pcppi/index.html

## Introduction

*Penicillium expansum* is a devastating phytopathogen that causes decay in crop plants during post-harvest handling and storage. This pathogen has a broad and variable host range, including apple, citrus fruit, pear, tomato, kiwifruit, maize and rice (1). As *P. expansum* exhibits physiological tolerance to many adverse environmental conditions, it can easily infect wounds and quickly spread through decaying tissue during storage and subsequent processing stage. Blue mold decay, resulting from *P. expansum* infection, causes significant agricultural losses worldwide, which is responsible for a major economic problem. Moreover, mycotoxins produced by molds can be deleterious to human health (2). Therefore, controls of blue mold and patulin contamination in major crops (in particular pome fruits) have received much attention (3). With the purpose of reducing the use of chemical fungicides, it is necessary to understand the molecular mechanisms of fungal infection and develop eco-friendly strategies for controlling this global post-harvest disease (4).

The symptoms of blue mold decay caused by *P. expansum* are very similar across different crop hosts. This suggests that the mechanism of pathogenicity may have a common mechanism (5). Typically, the infection process includes the following steps: attachment to host tissue, secretion of plant cell wall degradation enzymes and exopolysaccharides, release of toxins, acquisition of iron and the production of effector proteins (6). In fact, protein–protein interactions (PPIs) form the regulatory networks that coordinate diverse cellular functions (7). Based on whether the interacting proteins are derived from the same or different species, PPIs are typically classified into two categories: intra-species PPIs, where the two interacting proteins are derived from the same species, and inter-species PPIs, where the two interacting proteins are produced from different species (8). Pathogen–crop PPIs, a class of inter-species PPIs, play crucial roles in initiating the infection process and inducing a plant defense response (9).

Recently, several genes (e.g. *GOX2*, *IDH*) have been reported to be involved in the initial signaling cascades that stimulate the growth of *P. expansum* on crops (10, 11). Although these enzymes may have a role in pathogenicity, there is evidence that more complex interactions are taking place during the initial stages of infection and diseases development (10). To facilitate an effective approach for studying such complex interactions, bioinformatics has been suggested and employed to study the protein interaction networks at a genome-wide scale (12). While some proteomic studies have identified inter-species PPIs in model organisms, only a few have focused on pathogen–plant interactions. Therefore, research on effector biology in *P. expansum* is rather scarce (Table 1). In the current study, we constructed an interactomic resource for *P. expansum* by identifying potential protein–protein interaction from experimentally verified interacting orthologs in pathogen–plant systems. Thus far, we obtained 439 904 non-redundant PPIs between *P. expansum* and seven crops including apple, kiwifruit, maize, pear, rice, strawberry and tomato. These interactions have been entirely verified by both interolog mapping and DDIs supporting. In order to facilitate the use of these datasets, we have developed a searchable database, the *Penicillium*-Crop Protein–Protein Interactions (PCPPI), and described it here. PCPPI features a user-friendly interface for database search, information browse and interaction visualization. This resource and related documents are freely accessible at http://bdg.hfut.edu.cn/pcppi/index.html.

**Table 1:** Summary of PPIs among different species

Database	Content	Comment	References
PHI-base	It stores information on 2875 genes, 4102 interactions, 110 host species, 160 pathogenic species and 181 diseases.	Not provide PPI datasets.	(13)
PHISTO	23 661 PHIs between human and 300 pathogen strains.	No plants are included.	(14)
HPIDB	It integrates experimental PPIs from several public databases.	Only 1% of the datasets involves plants.	(8)
CAPIH	It provides PPIs between human and three model organisms.	It is a human specific database.	(15)
XooNET	It contains 4538 proteins and 26 932 possible interactions.	It is a rice and *Xoo* specific database.	(6)
Human-*Plasmodium* system	516 PPIs between proteins from these two organisms.	It is a human specific database.	(16)
HoPaCl-DB	It provides 3585 experimentally validated interactions.	It is a human specific database.	(17)
PPIRA	It contains 3074 potential PPIs between 119 *R. solanacearum* and 1442 *A. thaliana* proteins.	It is an *A. thaliana* and *R. solanacearum* specific database.	(18)
VirHostNet 2.0	It is dedicated to the virus-host PPIs.	It is a human specific database.	(19)
VirusMINT	It stores over 5000 interactions involving more than 490 viral proteins.	It is a human specific database.	(20)

## Materials and Methods

**Figure 1:**
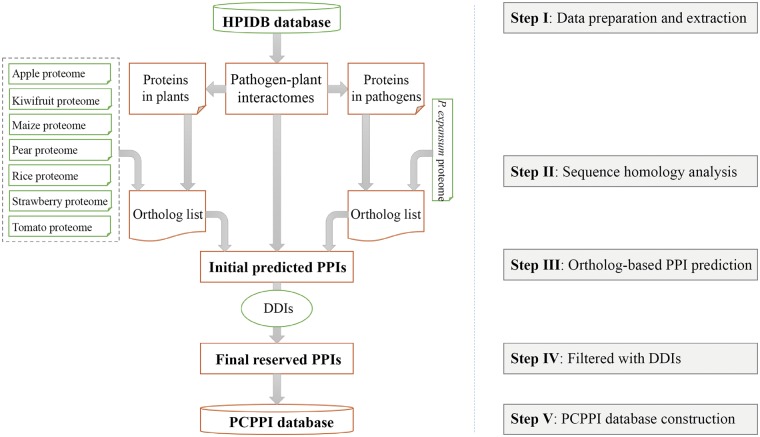
The flowchart for construction of PCPPI database.

The general process of data collection, extraction, integration, prediction, verification, and annotation is illustrated in Figure 1.

### Data collection and extraction

A total of 10 663 *P. expansum* protein sequences were downloaded from the EnsemblFungi database (release 29, http://fungi.ensembl.org/Penicillium_expansum/Info/Index) (21). The proteome datasets of apple (*Malus domestica*, 95 232 sequences) (22), kiwifruit (*Actinidia chinensis*, 39 761 sequences) (23), maize (*Zea mays*, 38 914 sequences) (24), pear (*Pyrus communis*, 45 217 sequences) (25), rice (*Oryza sativa*, 42 132 sequences) (26), strawberry (*Fragaria ananassa*, 45 377 sequences) (27) and tomato (*Solanum lycopersicum*, 33 785 sequences) (28) were obtained from the following databases: GDR (ftp://ftp.bioinfo.wsu.edu/species/Malus_x_domestica/Malus_x_domestica-genome.v3.0.a1/genes/), KIR (ftp://bdg.hfut.edu.cn/kiwi/Actinidia_chinensis), maizeGDB (http://ftp.maizegdb.org/MaizeGDB/FTP/maize_proteome/), GDR (ftp://ftp.bioinfo.wsu.edu/species/Pyrus_communis/Pcommunis-draft_genome.v1.0/genes/), EnsemblPlants (release 29, http://plants.ensembl.org/Oryza_sativa/Info/Index), GDR (https://www.rosaceae.org/node/4230473) and Solgenomics (release 2.4; ftp://ftp.solgenomics.net/tomato_genome/annotation/), respectively. Additionally, the interactome datasets of pathogen–host interactions were obtained from the HPIDB database (06-16-2015 release, 43 276 pairs; http://www.agbase.msstate.edu/hpi/main.html) (8). To implement the interolog method, we kept 361 interacting protein pairs from pathogen–plant interactions. Eventually, the DDI datasets were taken from the Database of Protein Domain Interactions (DOMINE) (09-2010 release, 26 219 pairs) (29).

### Sequence homology analysis

The protein sequences of pathogens and plants in the 361 interacting protein pairs were selected and integrated. Then, the TBLASTP query was performed to detect the homologous proteins with an *E*-value cutoff 1*E*-5 (30). While the homologous proteins in apple, kiwifruit, maize, pear, rice, strawberry and tomato were identified against plant proteins, the homologous proteins in *P. expansum* were analyzed with pathogen proteins.

### Prediction of potential PPIs

First, protein orthologs were mapped back onto the interactome datasets of pathogen–plant interactions. Any two proteins with orthologous mapping onto any one of these PPIs in pathogen–plant systems were recorded as a protein group with potential interaction. Then, each PPI was filtered by referring to the experimentally verified DDIs datasets, and only those PPIs carried with known DDIs were reserved.

### Annotation of protein information

After predicting the protein interactions, we collected all the interacting proteins involved and annotated them as follows: (i) the basic information, including protein name/synonyms, protein sequences, nucleotide sequences of the corresponding genes, MW (molecular weight) and theoretical PI, is annotated referring to UniProt Knowledgebase and GenBank; (ii) the biological function of proteins, such as the Pfam domain (31) and gene ontology (GO) annotation (32).

## Utility

### Database implementation

Currently, PCPPI operates on a Linux, Apache, MySQL and PHP (LAMP) stack. MySQL is used for storage, maintenance, and operation of the datasets. The front-end user interface is implemented in PHP, which is a popular scripting language designed for dynamic web development. A well-defined and packaged JavaScript called jQuery is used to improve website performance and enhance user experience. Also, the online BLAST program is employed for homology search and the Vis.js library (http://visjs.org/) is embedded within the web pages to display the predicted PPI datasets. Links, including Browse, Download and Documentation, are also provided in the navigation tool bar located on the top right of each web page.

### Query option

**Figure 2:**
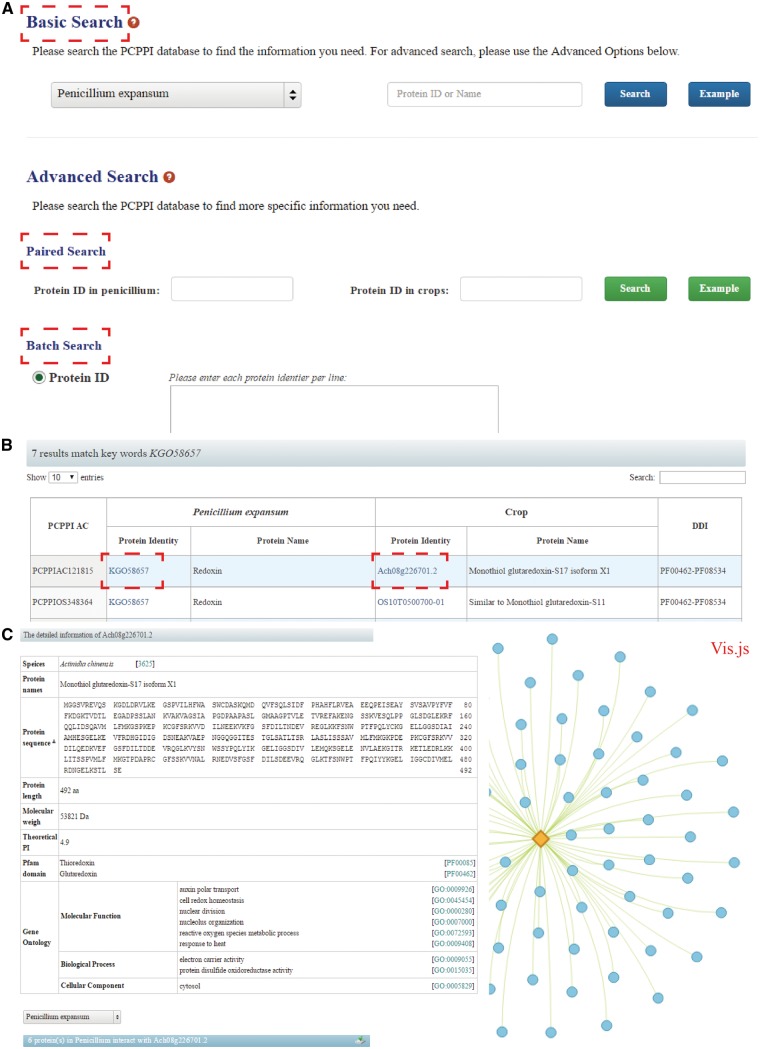
The search functions of PCPPI database. (A) Three search categories for querying. (B) The results are shown in a tabular format. Users can visualize the detailed information by clicking on the Proteins ID in *P. expansum* and/or crops. (C) The detailed information for each interacting protein.

PCPPI was developed in an easy-to-use mode. To access the database content, three search categories are provided: (1) Basic Search; (2) Paired Search; and (3) Batch Search (Figure 2A).

The basic search option provides an interface for querying the PCPPI with accession numbers (identifiers) or the keywords of gene/protein names. The full name and abbreviation are both feasible, where they are automatically normalized with synonyms. The species should be firstly specified by choosing their respective names in dropdown list.

The paired search options are accession number centric. Users can find the potential protein–protein interactions with the identifiers. The identifier of proteins in *P. expansum* should be typed in the left text box, and the identifier of proteins in crops should be entered in the right box. Records will be found only if these two proteins interact with each other.

The batch search option allows users to input a list of protein identifiers, or upload a file. Before submitting, the algorithms should be specified by selecting the setting ‘Show only pair wise interactions between the specified proteins’ or ‘Show all interactions involving specified proteins’.

The Paired Search and Batch Search are provided in the Advanced Search column.

### Detailed information

After searching, the results are displayed in a tabular format, containing PCPPI AC, Protein ID (in *P. expansum*), Protein name (in *P. expansum*), Protein ID (in crops), Protein name (in crops), DDI annotation (Figure 2B). From this table, users can browse the detailed information for each protein by clicking on the target Protein ID. Briefly, Protein names, Protein sequence and length, Molecular weight (MW), Theoretical PI, Pfam domain and GO annotation are presented in the detailed description of each interacting protein (Figure 2C). Access to known databases' interpretation of corresponding descriptions is also provided. In addition, the Vis.js library, a web browser applet integrated into the web pages, is used to visualize and analyze the involved protein interactions (edges) and the corresponding members (nodes), where proteins are graphed as nodes (one dimension) and interactions are graphed as edges (two dimension). It is hoped that this information will effectively aid users exploring the relationship between the proteins of interest. Finally, users can interact with the network and save the results in a text format.

## Results

### Building the interactome and data statistics

PPIs, which play essential roles in metabolic pathways and signal transduction, were identified based on the assumption that evolutionarily conserved proteins would form conserved interactions. This process is well known as interaction ortholog mapping and served as a proven and effective method for predicting interactomes (33). Over the years, it has been successfully applied within many model species, such as yeast (34), Arabidopsis (35), rice (36), *Brassica rapa* (37) and tomato (38). In this work, a total of 245 proteins responsible for pathogenicity in *P. expansum* have been predicted by using the orthologous mapping method. Meanwhile, 740–2270 proteins in diverse hosts that interact with the pathogenic proteins have been identified (Table 2). Finally, an easy-to-use and affordable web-based resource for analyzing protein interaction networks in pathogen–plant systems was developed.

**Table 2:** Summary of PPIs and proteins in different species

Species name	Common name	Number of PPIs	Number of proteins
*Malus domestica*	Apple	100 120	2270
*Actinidia chinensis*	Kiwifruit	51 538	1196
*Zea mays*	Maize	63 922	1347
*Pyrus communis*	Pear	75 055	1608
*Oryza sativa*	Rice	65 140	1425
*Fragaria ananassa*	Strawberry	33 240	740
*Solanum lycopersicum*	Tomato	50 889	1080
*Penicillium expansum*^a^	Blue mold	439 904^a^	245

a It includes the PPI datasets of *Penicillium expansum* interacting with the seven crops above.

**Figure 3:**
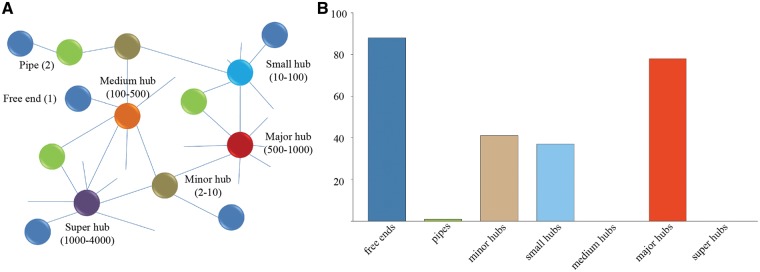
(A) Different categories of proteins classified according to our previous study. (B) Frequency distribution of the different categories in *P. expansum*. Value is the average of protein interactions from seven species.

In order to analyze the composition and topology of the constructed interactome, proteins of *P. exponsum* in the interaction networks were classified into free ends (with only one interaction), pipes (two interactions) and hubs (multiple interactions) of different sizes (minor hubs, small hubs, medium hubs, major hubs and super hubs) according to our previous work (Figure 3A) (38). As displayed in Figure 3B, the hub distribution shows an uneven distribution as a great deal of proteins belongs to the categories of free ends and major hubs.

Furthermore, the proteins in free ends and major hubs were assigned to molecular function and biological process categories according to the GO annotation from the Gene Ontology Consortium (Table 3). The results suggest that the proteins in major hubs are significantly enriched for protein kinase activity and mitogen-activated protein kinase (MAPK) cascade that could be activated by different stimuli in plant growth and defense (Hypergometric test) (39). Additionally, the proteins in free ends tend to possess oxidation–reduction process and electron carrier activity. As expected, these enrichments are generally consistent with the studies utilizing 2D-DIGE proteomics, which found that a majority of proteins are involved in protein metabolism, redox homeostasis and metabolic processes (40).

**Table 3:** The enrichment (Hypergometric test) and frequency of each GO term annotation (biological process and molecular function) for free ends and major hubs were calculated

GO term	Description	Free ends		Major hubs	
		Frequency	*P* value	Frequency	*P* value
*The category of biological process*					
GO:0000165	MAPK cascade	–	–	0.0513	3.27E−08
GO:0006464	Cellular protein modification process	0.0235	0.0058	–	–
GO:0006468	Protein phosphorylation	–	–	0.5769	7.90E−67
GO:0006511	Ubiquitin-dependent protein catabolic process	0.1059	5.12E−10	–	–
GO:0016310	Phosphorylation	–	–	0.3205	4.58E−26
GO:0043044	ATP-dependent chromatin remodeling	0.0118	0.015	–	–
GO:0055114	Oxidation–reduction process	0.5882	2.73E−24	–	–
*The category of molecular function*					
GO:0004672	Protein kinase activity	−	−	0.9744	9.28E−136
GO:0005524	ATP binding	0.2471	2.24E−09	−	−
GO:0008233	Peptidase activity	0.0706	0.0014	−	−
GO:0016491	Oxidoreductase activity	0.5765	1.47E−34	−	−
GO:0016538	Cyclin-dependent protein serine/threonine kinase regulator activity	−	−	0.0128	0.014
GO:0019948	SUMO activating enzyme activity	0.0118	0.016	−	−
GO:0036459	Ubiquitinyl hydrolase activity	0.035	0.00012	−	−
GO:0051903	S-(hydroxymethyl)glutathione dehydrogenase activity	0.0118	0.016	−	−

### Evolutionary conservation of domain–domain interactions

Generally, conserved domains are the main functional and structural units of proteins and play important roles in PPIs by forming interactions in diverse ways (heterotypic or homotypic) (41). DDIs, acting as the building blocks of PPIs, are more conserved than the PPIs themselves (42). In the present study, all of the final reserved PPIs were verified by DDIs supporting. This will not only increase the confidence of our predictions, but also provide more detailed information about which domains are potentially involved in mediating the binding of two interacting proteins. Notably, the LysM domain (Pfam: PF01476) is known to be involved in interacting with a variety of enzymes and has a general peptidoglycan binding function (43). Proteins carrying LysM domain in plants have been implicated in the recognition of carbohydrate patterns commonly associated with pathogen surfaces and thus play a role in the response to pathogen infection (44). As a widely distributed protein motif, there are total 106 domains that interact with the LysM domain in the datasets of experimentally observed DDIs (Supplementary Table S1) (29). Furthermore, a total of 1869 PPIs in 35 proteins were identified in the LysM datasets in PCPPI (Supplementary Tables S2 and S3). Functional annotation revealed that these 35 proteins are almost the LysM receptor kinases, which play critical roles in chitin elicitor signaling. While kinases could aid in restricting bacterial/fungal growth on plants, plant pathogens may utilize effector proteins to degrade kinases and thus block defense responses (45).

### Accuracy evaluation

The datasets of PCPPI are predicted from orthologous interactions and further filtered based on the DDI information, which have been entirely well studied through the experiments. To confirm the feasibility of the integrative approach that DDI filtration could improve the precision of prediction, an independent dataset of 361 protein pairs was randomly selected from the 360 249 455 possible interactions between *P. expansum* and tomato, and then used as the testing data (Supplementary Table S4). Correspondingly, the 361 protein interactions were referred as the experimental data. Subsequently, the involved DDI information was identified and analyzed between each protein group. Finally, 56 PPIs with known DDIs were found in the experimental data (Supplementary Table S5), which is significantly higher than the number of eight for the testing data (*P*-value < 1*E*-3, *T*-test; Supplementary Table S4). This result undoubtedly confirms the usefulness of DDI information for improving the accuracy of our predictions.

Furthermore, the above analysis of GO annotations has presented the enriched biological themes and functional proteins that clustered in *P. exponsum*. Other than co-evolution, the proteins in crops commonly perform a given function together with the interacting proteins in *P. exponsum* as they cannot work individually (46). Therefore, investigating the sharing of GO terms between two interacting proteins could also support the reliability of our predicted datasets in PCPPI. In the present study, the semantic relationships of GO terms in the biological process category were analyzed and calculated for the two proteins of each predicted PPI according to the method described by Lin (47). The highest semantic similarity for every PPI was retained and the final average values of the interactomes between *P. exponsum* and apple, kiwifruit, maize, pear, rice, strawberry and tomato were 0.793, 0.638, 0.737, 0.867, 0.868, 0.543 and 0.830, respectively. These values are all significantly higher than a value of 0.10 which would be expected for a randomized connection (*P*-value < 1*E*-10, Wilcoxon test). Therefore, the significant overlap of the identified GO terms between *P. exponsum* and crops greatly support the quality of the predictions generated in PCPPI as well.

## Conclusion

In summary, PCPPI is the first web database providing comprehensive information about predicted PPIs involved in the pathogenicity and virulence of *P. expansum* on crop plants. Identifying these interactions is helpful to elucidate the molecular mechanisms underlying fungal pathogenicity, signal transduction and metabolic pathways. These datasets could be used for both theorists and experimentalists to reassemble protein complexes, expand existing pathways, as well as discover potential new antibiotic targets. In order to provide up-to-date data on protein interactions, we will continue to collect and incorporate the latest datasets of PPIs and DDIs, and develop new methods to predict potential protein interactions. Moreover, we will reiterate the process of database structure and user interface design to enhance the web content and functionality.

## Supplementary Data

Supplementary data are available at *Database* Online.

## Funding

National Natural Science Foundation of China [31461143008 and 31471157]; the Joint NSFC-ISF Research Program, jointly funded by the National Natural Science Foundation of China and the Israel Science Foundation [1936/14]; Fundamental Research Funds for the Central Universities [JZ2016HGBZ1017]; National Science Fund for Distinguished Young Scholars [30825030]; Advanced Program of Doctoral Fund of Ministry of Education of China [20110181130009]; Key Project from the Government of Sichuan Province [2013NZ0014]; Key Project from the Government of Anhui Province [1401032006]. Funding for open access charge: Joint NSFC-ISF Research Program, jointly funded by the National Natural Science Foundation of China and the Israel Science Foundation [1936/14].

*Conflict of interest*. None declared.

## Supplementary Material

Supplementary Data
